# Efficacy of Articaine and Lidocaine for Buccal Infiltration of First Maxillary Molars with Symptomatic Irreversible Pulpitis: A Randomized Double-blinded Clinical Trial

**DOI:** 10.7508/iej.2016.02.001

**Published:** 2016-03-20

**Authors:** Hamid Reza Hosseini, Masoud Parirokh, Nouzar Nakhaee, Paul V. Abbott, Syamak Samani

**Affiliations:** a *Endodontic Department, Dental School, Khorasan Shomali University of Medical Sciences, Bojnord, Iran; *; b * Endodontology Research Center, Kerman University of Medical Sciences, Kerman, Iran; *; c * Neuroscience Research Center, Kerman, Iran; *; d * School of Dentistry, University of Western Australia, Perth, Australia; *; e *Endodontic Department, Dental School, Shahid Saddoghi University of Medical Sciences, Yazd, Iran*

**Keywords:** Articaine, Buccal Injection, Infiltration, Irreversible Pulpitis, Lidocaine, Maxillary Molar, Palatal Root, Root Length

## Abstract

**Introduction:**

The aim of the present study was to compare the efficacy of 2% lidocaine to 4% articaine in buccal infiltration of maxillary first molars with irreversible pulpitis. Moreover, the effect of root length on success of anesthesia irrespective of the type of anesthetic agent was assessed.

**Methods and Materials:**

Fifty patients suffering from painful maxillary first molars with irreversible pulpitis received an infiltration injection of either 4% articaine with 1:100000 epinephrine or 2% lidocaine with 1:80000 epinephrine. Each patient recorded their pain score in response to a cold test on a Heft-Parker visual analogue scale (VAS) before commencing the treatment, 5 min following injection, during access preparation, after pulp exposure and during root canal instrumentation. No or mild pain at any stage was considered a success. Data were analyzed using the multivariate logistic regression analysis, chi-square and t tests.

**Results:**

Finally, 47 out of 50 patients were eligible to be included in this study. The anesthetic success rates in the lidocaine and articaine groups were 56.52% and 66.67%, respectively and the difference was not significant (*P*=0.474). Irrespective of the anesthetic agent, the length of the palatal root (Odds Ratio=0.24, *P*=0.007) had an adverse effect on anesthetic success. There was an association between longer palatal root length and anesthetic failure.

**Conclusion:**

No significant difference was found between 2% lidocaine and 4% articaine in terms of anesthetic success in maxillary first molars with irreversible pulpitis. The length of the palatal root had a significant negative influence on anesthetic success.

## Introduction

pain control during and after root canal treatment is an important subject that has attracted considerable attention [1-8]. One of the most important steps during root canal treatment of teeth with irreversible pulpitis is to provide profound anesthesia in order to prevent pain perception during the procedure [9]. Numerous investigations have focused on assessing different devices, different anesthetic agents, and supplemental anesthetic techniques to increase the ability of clinicians to overcome pain during treatment and to provide higher success rates of anesthesia [9-22]. Most of these investigations have been performed on mandibular teeth due to the perception that achieving anesthesia in mandibular teeth is much more difficult than their maxillary counterparts [9]. 

Lidocaine is the most widely used dental anesthetic agent [23]. Articaine has been reported to be a superior anesthetic solution for infiltration injection. Several studies have compared the efficacy of articaine and lidocaine following injection in the maxillary molar region [10, 13, 24-27]. The results of two meta-analyses favored the effectiveness of articaine over lidocaine for infiltration injection [28, 29]. However, most previous investigations these solutions are crossover studies and only three studies compared them for maxillary first molars with irreversible pulpitis. These studies reported conflicting results regarding the efficacy of the two anesthetic solutions [10, 26, 27]. Two of them reported no significance different between lidocaine and articaine [26, 27], whereas the other one reported a significantly higher anesthetic success when articaine was used [10].

Anesthetic success may depend on several variables such as the pulp status, the presence of inflammation in the pulp, and whether any anesthetic supplemental technique is used [9]. Another variable that might influence the efficacy of anesthesia is the root length [30]. In theory, injection of the anesthetic agent more coronally than the location of root apex might adversely affect the success of anesthesia. However, no study is available regarding the effect of root length on anesthesia of maxillary molars. Therefore, the aims of this study were firstly to compare the anesthetic efficacy of 2% lidocaine with 1:80000 epinephrine and 4% articaine with 1:100000 epinephrine on asymptomatic maxillary molars with irreversible pulpitis and secondly, to investigate the effect of root length on the efficacy of anesthesia irrespective of the type of anesthetic agent.

## Materials and Methods

This study was approved by the Ethics Committee of Kerman University of Medical Sciences in Iran (KA.92.114) and Iranian Registry of Clinical Trials (ID: IRCT201204302016N5). The sample size calculation, which was based on type I error of 0.05 and power of 0.8, indicated that ideally a sample size of 25 in each group would be required. 

The inclusion criteria used in this study were: healthy adult patients over 18 years of age having a ﬁrst maxillary molar with asymptomatic irreversible pulpitis and normal periapical radiographic appearance. Pulp vitality was determined by a positive response to EPT (SybronEndo, Glendora, CA) and cold tests (Roeko Endo Frost, Roeko, Hangenav, Germany) and a diagnosis of asymptomatic irreversible pulpitis was made if a prolonged response to cold (more than 10 sec) was noted. 

The exclusion were the presence of systemic disorders, any known sensitivity to either 2% lidocaine or 4% articaine or epinephrine, the widening of periodontal ligament space, the presence of a periapical radiolucency, pregnancy, using any type of analgesics 12 h before the treatment, moderate to severe spontaneous pain, tenderness to percussion and having a tooth not suitable for simple restorative treatment because of extensive caries or periodontal problems.

Fifty patients were eligible to participate in this prospective, randomized double-blind study. All patients were treated in the postgraduate clinic of the Endodontic Department of Kerman Dental School in Iran from Sep 2013 to Jan 2014. All subjects signed an informed consent form in which the nature of the procedure and the possible discomforts and risks were fully described.

The patients were randomly divided into two groups of 25 patients each. In order to randomize the patients, the number of patients in each group were written on paper and kept in a sealed box. The practitioner who administrated the local anesthesia chose one of the papers and based on the number, the patient was assigned to one of the groups. Another practitioner did the cold test and then prepared the access cavity after administration of the anesthetic agent. Therefore neither the second practitioner nor the patients were aware of the type of anesthetic agents used. 

Patients in group 1 were given a buccal infiltration injection of 1.8 mL 2% lidocaine with 1:80000 epinephrine (Persocaine-E, Daru Pakhsh Pharmaceutical Mfg. Co., Tehran, Iran) and patients in group 2 had the same injection but with 1.8 mL of 4% articaine with 1:100000 epinephrine (Artinibsa, Inibsa, Barcelona, Spain).

Before injection, the patients were asked to rate their pain using a Heft-Parker visual analog pain scale (VAS) after the cold test. The VAS scores were divided into four categories. No pain corresponded to 0 mm, mild pain was deﬁned as being in the range of 0< and <54 mm, moderate pain was deﬁned as being <54 and <114, and severe pain was deﬁned as being ≥114 mm.

After applying topical anesthesia (20% Benzocaine; Premier, Philadelphia, PA, USA) to the site of the injection, a standard maxillary buccal infiltration was administered using an aspirating syringe with a side-loading cartridge system (Dena Instruments, Forgeman Instruments Co., Pakistan) and a 27-gauge 25 mm needle (C-K ject, CK Dental, Kor-Kyungji-do, Korea). The injection site was between the estimated location of mesiobuccal and distobuccal root apices of the maxillary first molar. The needle was gently placed into the alveolar mucosa with the bevel toward the alveolar bone and penetrated until it was estimated to be above the apices of the buccal roots of the teeth. All injections were given by the same clinician. Five min after administering anesthesia, the teeth were again tested with the same cold pulp sensibility test and the patients were asked to rate their pain using the Heft-Parker VAS [31].

The teeth were then isolated with rubber dam and an endodontic access cavity was prepared. The patients were informed to rate any pain that they experienced during each step of access cavity preparation including cutting within dentin, entering the pulp chamber or during root canal instrumentation. Absence of pain or mild discomfort was considered as success whereas moderate or severe pain was considered as failure of anesthesia. In case of sensitivity to the cold test before starting the access cavity or if pain was reported at any stage during treatment, another method of anesthesia (palatal infiltration, intra-ligamentary, and intra-pulpal injections as supplemental techniques) was employed to overcome the patients’ discomfort. 

In each tooth, for root canal measurement, at first the coronal third of all root canals were enlarged with #2 Gates Glidden drills (Mani, Tochigi, Japan) and then the root canal length was measured with Root ZX apex locator (J. Morita Corporation, Kyoto, Japan) with a #10 K-file (Mani, Tochigi, Japan). Then the root canal was instrumented until at least #15 K-type file can be inserted to the full working length as measured by the apex locator. Then a periapical radiography was taken from the tooth. If the measurement was within ±1 mm from the radiographic apex, the file was adjusted into the root canal and another radiography was taken. Meanwhile, the root canal was again measured with the apex locator. After establishing the measurement, root canal instrumentation were completed with RaCe rotary instruments (FKG Dentaire, La Chaux-de-Fonds, Switzerland) with the same sequence of a previous investigations [1] and master gutta-percha cones were inserted into the root canals and another periapical radiograph was taken. 

Comparisons were done using the t-test for continuous variables and chi-square or Fischer’s exact test for categorical data. Multivariate logistic regression analysis was performed to identify variables that could be significant predictors of success in treatment. *P-values* less than 0.05 were considered as significant.

## Results

At the final stage of the treatment, 47 out of 50 patients were found to be eligible for the study. Two patients (one each from the articaine and the lidocaine groups) were excluded because the pulp was not exposed while one patient from the lidocaine group was excluded because the pulp was partially necrotized noted after pulp exposure. No side effect was found among the patients after administration of anesthesia.

There was no significant difference in gender and age between the two groups (*P*=0.45 and *P*=0.90, respectively). Final success of anesthesia was 56.52% for the lidocaine group and 66.67% for the articaine group (Table 1). There was no significant difference between the groups (*P*=0.47).

Analysis of the distobuccal and mesiobuccal root lengths showed no significant influence on the efficacy of anesthesia irrespective of which anesthetic agent was used (*P*=0.72 and *P*=0.89, respectively) (Table 2). In contrast, the palatal root length had a significant influence on the efficacy of anesthesia meaning that shorter palatal roots showed significantly higher successful anesthesia than longer roots [Odds Ratio (OR)=0.24, *P*=0.007]. 

**Table 1:** Number (percentage) of success and failure at various steps during access cavity preparation and root canal instrumentation

	**Success N (%)**		**Failure N (%)**		***P-*** **Value**
	**Lidocaine**	**Articaine**	**Lidocaine**	**Articaine**	
**5 min**	23 (100)	22 (91.7)	0 (0)	2 (8.3)	0.489
**Dentin**	21 (91.3)	22 (100)	2 (8.7)	0	0.489
**Pulp**	17 (80.95)	21 (95.5)	4 (19.05)	1 (4.5)	0.185
**Filing**	13 (76.47)	16 (76.2)	4 (23.53)	5 (23.8)	1.000
**Final Success**	13 (56.52)	16 (66.67)	10 (43.48)	8 (33.33)	0.474

**Table 2:** Factors associated with final success of anesthesia (OR=odds ratio

		**Adjusted OR**	**CI 95%**	***P*** **-value**
**Age**		1.20	0.99-1.45	0.058
**Sex**	**Male**	1	--	--
	**Female**	0.097	0.01-1.64	0.106
**Group**	**Lidocaine**	1	--	--
	**Articaine**	2.12	0.34-13.14	0.418
**Root**	**Mesiobuccal**	1.07	0.38-2.98	0.893
	**Distobuccal**	1.19	0.45-3.15	0.723
	**Palatal**	0.24	0.08-0.67	0.007

## Discussion

The results of the present study showed no significant difference between 4% articaine and 2% lidocaine on anesthetic success following an infiltration injection for maxillary first molars with irreversible pulpitis (*P*=0.47). In addition, the palatal root length significantly affected the anesthetic success, whereas the mesiobuccal and distobuccal root lengths had no significant influence on anesthesia, irrespective of the anesthetic agent used.

The onset of anesthesia is an important issue for anesthesia success evaluation. The onset of anesthesia in inferior alveolar nerve block and infiltration injection are different and the letter technique provide quicker anesthesia [3, 9]. The onset of anesthesia in maxillary teeth is usually achieved within 5-7 min after administration of anesthesia [10, 25, 26, 32]. Therefore, in the present study, a cold pulp sensibility test was performed 5 min after administration of anesthesia to initially test the effectiveness of the injection.

In theory, during infiltration injection of maxillary molars the needle should penetrate deep enough inside the buccal tissues to deposit the anesthetic agent as close as possible to the root apex to increase anesthetic success [30]. This method is relatively simple for single-rooted teeth. However, for teeth with multiple roots such as maxillary first molars, the effect of root length on the efficacy of anesthesia has not been investigated previously. In the present study, the mesiobuccal and distobuccal root lengths showed no significant influence on the anesthetic success (*P*=0.893 and *P*=0.723, respectively), but the palatal root length did show a significant influence on the success of injection (OR=0.24, *P*=0.007). This is consistent with other studies that have reported a single buccal infiltration may not be effective for anesthetizing the palatal roots of maxillary molars [33, 34]. The major reason may be the distance between the site of injection and the root apex of the palatal root [30]. Thus, it is likely that maxillary molars with long or more divergent palatal roots may present anesthetic difficulties since the root apex is more distant from the injection site. Hence, from the clinical point of view, it can be recommended that if the pre-operative periapical radiography shows a molar tooth with long roots, then a supplemental injection should be considered before commencing access cavity preparation.

Several investigations have been performed to evaluate anesthesia success for maxillary molars [10, 25-27, 33-42]. Previous investigations compared the efficacy of single buccal infiltration injections versus both buccal and palatal injections and have reported no significant difference [40, 41]. Since palatal injection provokes pain and discomfort for the patient [9] in the present study only a single buccal injection of the tested anesthetic agents were used. 

The results of the present study were in contrast with the results of two previously published meta-analyses that compared the efficacy of articaine with lidocaine following infiltration injection [28, 29]. However, both meta-analyses had two major shortcomings. Firstly, these analyses combined data from studies investigating anesthesia of irreversible pulpitis and normal pulps. It has been shown that the risk of anesthesia failure in teeth with irreversible pulpitis is much higher than those with normal pulps. Therefore including data from both types of studies for meta-analysis is inappropriate [9]. Secondly, both meta-analyses included studies that used articaine for mandibular and maxillary infiltration injections. From the anatomical stand point, the cortical plate at the molar region of the mandible is much thicker than in the maxilla [30] and this can inhibit infiltration of the anesthetic agent. Therefore, including studies of teeth from both arches and comparing articaine with lidocaine in a meta-analysis may lead to bias in the results. In addition, the results of the present study were in accordance with two other investigations that showed no significant difference between the efficacy of articaine with lidocaine when anesthetizing maxillary first molars with irreversible pulpitis [26, 27].

The pulpal status and the diagnosis of pulp and periapical disease at the time of procedure may be important issues in success rate of anesthesia [9]. In the present study, the pulp status was one of the inclusion criteria and the presence of bleeding upon gaining access to the pulp was essential for the tooth to be included in the study [43]. Therefore, one patient in the lidocaine group was excluded because partial pulp necrosis was noted once the access cavity was prepared. Based on the definition of various stages of the pulp and the periapical diseases, patients may present with symptomatic or asymptomatic irreversible pulpitis [44, 45]. In the present study, only teeth with asymptomatic irreversible pulpitis was included. The reason is the conflicting results of previous investigations when premedication with NSAIDs was used for evaluating their effect on anesthesia success [11, 46-49]. In fact, previous investigations showed conflicting results regarding pulpal anesthesia success rate in patients with and without spontaneous pain when premedication with NSAIDs was used [11, 46-49]. The investigations that included patients with spontaneous pain reported no significant difference in anesthesia success when the patients were used premedication with NSAIDs [46, 48, 49], whereas the investigations that included patients without spontaneous pain reported significantly higher success when the NSAIDs were used for premedication [11, 47]. As the same bias may influence the success rate of anesthesia again, in the present study, only patients that had irreversible pulpitis but no spontaneous pain were included. Investigators should notice the possible bias and design their future research with careful inclusion criteria for various conditions of pulp and periapical diseases in order to provide more reliable results.

In the present study, based on the multivariate logistic regression analysis an association between longer palatal root and failure to anesthesia was found. However, if someone want to provide a cut-off point for the palatal root length, need to do another research with only one anesthetic agent to provide sensitivity and specificity. Right now the authors are working on a study to find out the cut-off point using ROC analysis and calculating sensitivity and specificity.

## Conclusion

The type of anesthetic solution had no significant influence on the success rate of anesthesia with articaine and lidocaine being similarly effective. However, the length of the palatal root was shown to adversely affect the success of anesthesia irrespective of the anesthetic agent used.
